# Early Oxidative Stress Response in Patients with Severe Aortic Stenosis Undergoing Transcatheter and Surgical Aortic Valve Replacement: A Transatlantic Study

**DOI:** 10.1155/2019/6217837

**Published:** 2019-11-13

**Authors:** Michael Mahmoudi, Juan Guillermo Gormaz, Marcia Erazo, Michael Howard, Cristian Baeza, Martin Feelisch, Nick Curzen, Bartosz Olechowski, Bernadette Fernandez, Magdalena Minnion, Monika Mikus-Lelinska, Mia Meiss, Laurie Lau, Nicolas Valls, Abraham I. J. Gajardo, Amy Rivotta, Rodrigo Carrasco, Gabriel Cavada, Maria Jesus Vergara, Gabriel Maluenda

**Affiliations:** ^1^Faculty of Medicine, University of Southampton, UK; ^2^Faculty of Medicine, Universidad del Desarrollo, Clinica Alemana, Santiago, Chile; ^3^Faculty of Medicine, University of Chile, San Borja Arriaran Hospital, Santiago, Chile

## Abstract

Myocardial ischemia/reperfusion-related oxidative stress as a result of cardiopulmonary bypass is thought to contribute to the adverse clinical outcomes following surgical aortic valve replacement (SAVR). Although the acute response following this procedure has been well characterized, much less is known about the nature and extent of oxidative stress induced by the transcatheter aortic valve replacement (TAVR) procedure. We therefore sought to examine and directly compare the oxidative stress response in patients undergoing TAVR and SAVR. A total of 60 patients were prospectively enrolled in this exploratory study, 38 patients undergoing TAVR and 22 patients SAVR. Reduced and oxidized glutathione (GSH, GSSG) in red blood cells as well as the ferric-reducing ability of plasma (FRAP) and plasma concentrations of 8-isoprostanes were measured at baseline (S1), during early reperfusion (S2), and 6-8 hours (S3) following aortic valve replacement (AVR). TAVR and SAVR were successful in all patients. Patients undergoing TAVR were older (79.3 ± 9.5 vs. 74.2 ± 4.1 years; *P* < 0.01) and had a higher mean STS risk score (6.6 ± 4.8 vs. 3.2 ± 3.0; *P* < 0.001) than patients undergoing SAVR. At baseline, FRAP and 8-isoprostane plasma concentrations were similar between the two groups, but erythrocytic GSH concentrations were significantly lower in the TAVR group. After AVR, FRAP was markedly higher in the TAVR group, whereas 8-isoprostane concentrations were significantly elevated in the SAVR group. In conclusion, TAVR appears not to cause acute oxidative stress and may even improve the antioxidant capacity in the extracellular compartment.

## 1. Introduction

Myocardial ischemia/reperfusion injury (MRI) related to cardiopulmonary bypass has been linked to adverse clinical outcomes following cardiac surgery [1–4]. Changes in reactive oxygen species (ROS) following surgical aortic valve replacement (SAVR) have been well documented in the literature [3]. Furthermore, preoperative ROS biomarkers such as malondialdehyde, a reactive breakdown product of lipid oxidation, have been shown to be predictors of adverse outcomes at 30-day and 1-year follow-up [5]. In contrast to SAVR, transcatheter aortic valve replacement (TAVR) is associated with shorter duration of myocardial ischemia and hypotension and may be associated with a lower degree of MRI. Apart from a single study using a new electrochemical technique to assess redox status in serum [6], which suggests that TAVR may be associated with lower oxidative stress as compared to SAVR, pertinent additional information is sparse [7]. However, the regulation of redox status in the intracellular compartment differs considerably from that in the extracellular space [8], and physiological processes are not governed by simple electrochemical potentials [9, 10]. Moreover, “oxidative stress” is an open concept with many contributing factors, the significance of which have not been established. The aim of the present study was to describe the early oxidative stress response in the blood of patients undergoing TAVR and compare them with a group of patients undergoing SAVR by applying established biochemical readouts of cellular and extracellular redox status.

## 2. Methods

### 2.1. Study Design

This was a prospective, observational, exploratory study designed to compare two series of patients with severe symptomatic aortic stenosis (AS) undergoing either TAVR (*n* = 38) or SAVR (*n* = 22). The study was conducted at the San Borja Arriaran Cardiovascular Center (Santiago, Chile), Clinica Alemana (Santiago, Chile), and the University Hospital Southampton NHS Foundation Trust (Southampton, UK). All patients gave written informed consent, and the study was conducted under Ethics Committee Board approval (Central Metropolitan Health Service Ethical Committee, Santiago, Chile, Project ID: 378/14; RES Committee North West Liverpool East, UK, IRAS Project ID: 206946). The study is registered at ClinicalTrials.gov (NCT02841917).

### 2.2. Eligibility Criteria for TAVR

Severe AS was defined by transthoracic echocardiography as aortic valve area < 1 cm^2^ or index valve area < 0.8 cm^2^/m^2^ in the presence of mean aortic gradient > 40 mmHg or peak velocity > 4 m/s. The local Heart Team assessed each candidate with severe AS according to the clinical background and imaging. Consideration was given in each case to the estimated surgical risk, including such features as severe comorbidities, advanced age, frailty, or thoracic anatomy unfavorable for SAVR. Society of Thoracic Surgeons (STS) score > 8% for mortality defined patients at a high risk for SAVR. There were no age restrictions for TAVR. Access site, either transfemoral or transapical, was determined according to the Heart Team recommendation.

### 2.3. Eligibility Criteria for SAVR

Patients with severe symptomatic AS undergoing SAVR served as the control group. These patients fulfilled the same echocardiographic criteria as patients undergoing TAVR but were considered to be at a lower surgical risk by the Heart Team. There were no age restrictions for SAVR.

### 2.4. Baseline Evaluation

The following studies were performed prior to the Heart Team evaluation: (1) complete blood count, coagulation tests, serum biochemistry, and liver function tests; (2) transthoracic echocardiogram; (3) coronary angiography, and (4) CT angiographic assessment of the ascending aorta, the thoracic aorta, and the aorto-iliofemoral tree.

### 2.5. Oxidative Stress Measurements

Peripheral venous blood was collected, using EDTA, heparin, and serum vacutainers, prior to the patients being transferred to the operating theatre for their procedure (S1), within 10 minutes of completion of SAVR/TAVR (S2) and 6-8 hours post SAVR/TAVR (S3). Samples were immediately subjected to centrifugation for separation into plasma, serum, and red blood cells and stored at -80°C. Samples were analyzed for (1) antioxidant potential in the extracellular compartment using the “ferric-reducing ability of plasma” (FRAP) [11], (2) intracellular redox status by measurement of reduced (GSH) and oxidized glutathione (GSSG) in red blood cells [12], and (3) lipid oxidation by measurement of plasma concentrations of 8-isoprostane (8-epi-prostaglandin-F_2*α*_) using a commercial assay kit (Cayman Chemical).

### 2.6. Statistical Analysis

Continuous variables are expressed as the mean ± SD. Categorical variables are expressed as frequencies and percentages. Baseline characteristics and postprocedural differences were compared using the chi-square independence test for categorical variables and the Student *t*-test for continuous variables. Intratime values per group were compared through regression analysis adjusted by the STS score. Intragroup changes in oxidative stress biomarkers were analyzed in terms of the slope of the trend. A mixed model was applied to evaluate the potential effects of red blood cell transfusion and procedural time variables, including “procedural time,” “fluoroscopic time,” “rapid-pacing time,” and “cardiopulmonary by-pass time,” on ROS following AVR. Statistical significance was assumed at *P* < 0.05. Statistical analyses were performed using Stata version 15® (StataCorp LLC, Texas, USA).

## 3. Results

The baseline clinical characteristics are summarized in Table 1. As compared to the SAVR group, female gender, older age, diabetes mellitus, smoking history, previous myocardial infarction, coronary artery disease, chronic renal impairment, and history of atrial fibrillation were more common in the TAVR group. The TAVR group had a higher risk profile than the SAVR group (mean STS score 6.6 ± 4.8 vs. 3.2 ± 3.0; *P* < 0.001; mean Log Euroscore 2 12.7 ± 8.8 vs. 5.1 ± 7.9; *P* < 0.001).

Baseline echocardiographic findings are summarized in Table 2. All patients underwent successful TAVR and SAVR. Procedural details are described in Table 3. One patient developed cardiac tamponade during TAVR due to pacing wire-induced right ventricular perforation, which was treated with pericardiocentesis.

**Figure 1:**
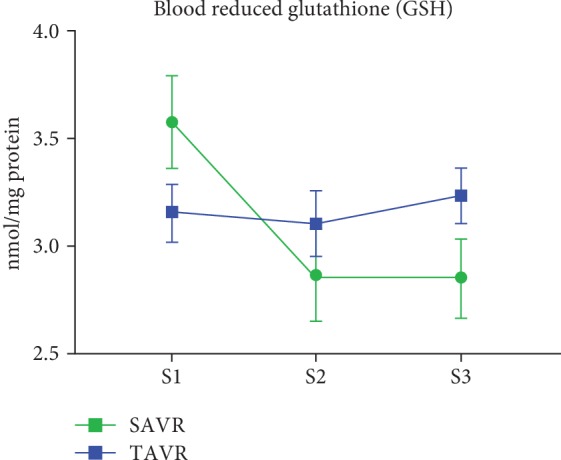
Blood reduced glutathione following aortic valve replacement. The reduced glutathione levels in red blood cells dropped significantly following SAVR whereas no change was observed after TAVR. Abbreviations: S1: sample 1; S2: sample 2; S3: sample 3.

**Figure 2:**
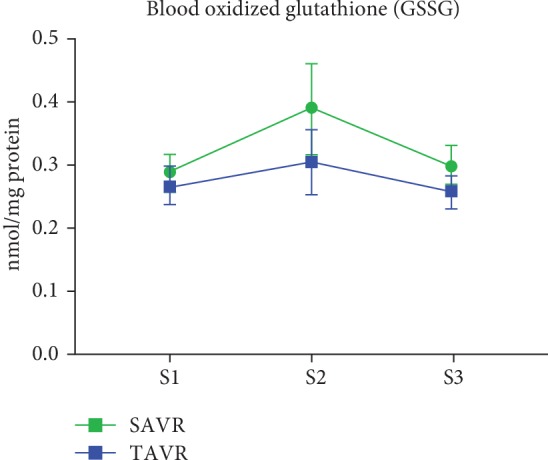
Blood oxidized glutathione following aortic valve replacement. Changes in oxidized glutathione concentrations in red blood cells were similar among patients undergoing SAVR or TAVR. Abbreviations: S1: sample 1; S2: sample 2; S3: sample 3.

**Figure 3:**
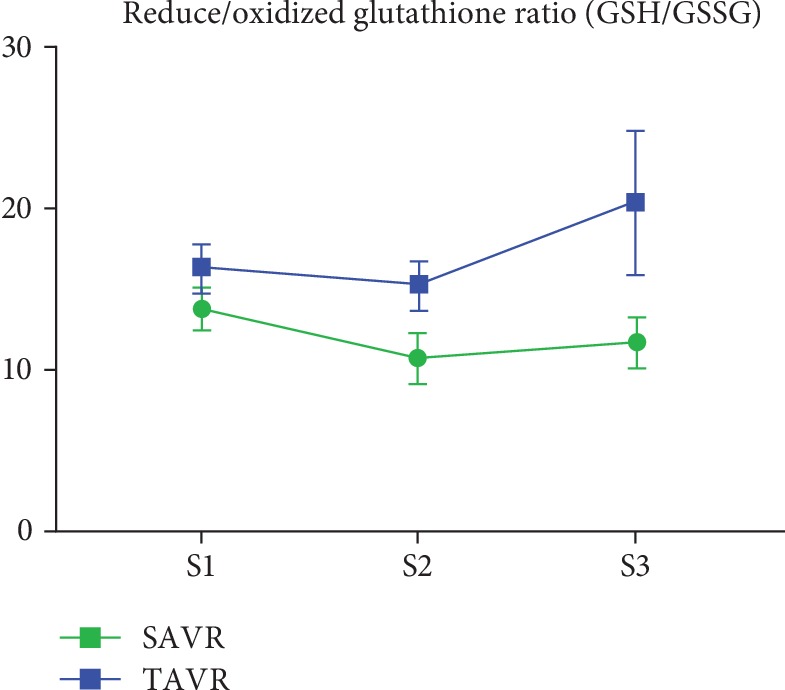
Ratio of blood reduced over oxidized glutathione following aortic valve replacement. Changes in reduced/oxidized glutathione ratio in red blood cells were similar among patients undergoing SAVR or TAVR. Abbreviations: S1: sample 1; S2: sample 2; S3: sample 3.

**Figure 4:**
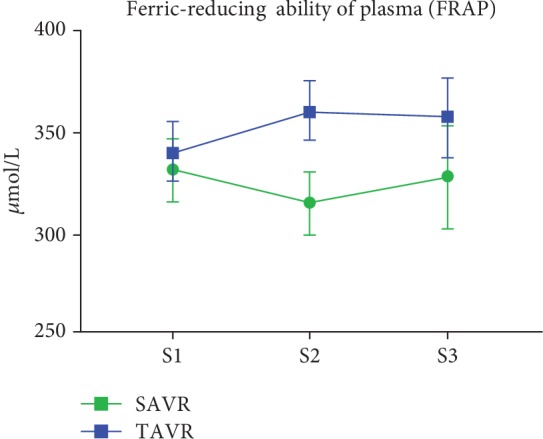
Ferric-reducing ability of plasma following aortic valve replacement. The ferric-reducing antioxidant power of the plasma increased significantly following TAVR whereas no change was observed after SAVR. Abbreviations: S1: sample 1; S2: sample 2; S3: sample 3.

**Figure 5:**
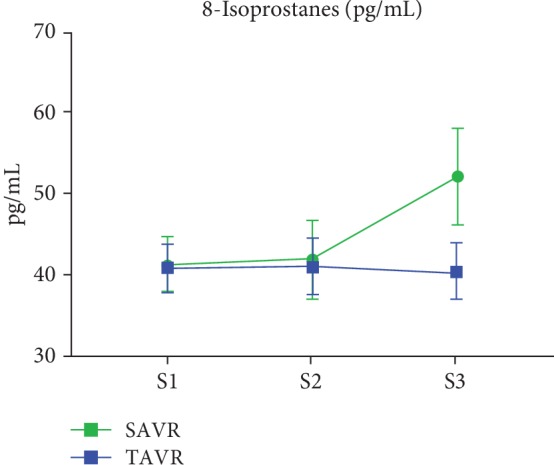
Plasmatic concentrations of 8-isoprostanes following aortic valve replacement. The 8-isoprostane levels in plasma increased significantly following SAVR whereas no change was observed after SAVR. Abbreviations: S1: sample 1; S2: sample 2; S3: sample 3.

Absolute values and changes in oxidative stress biomarkers are summarized in Table 4 and Figures 1–5. At baseline, the TAVR group had significantly lower GSH concentrations, similar concentrations of GSSG, and similar GSH/GSSG ratios. Intragroup changes showed a significant drop in GSH concentration following SAVR, whereas no significant changes in concentrations of GSSG or GSH/GSSG ratio occurred in the two groups (Figures 1–3). Following AVR, the concentrations of GSH and GSSG and the GSH/GSSG ratio were similar in the two groups. Baseline FRAP and 8-isoprostane concentrations were similar between the two groups. FRAP levels showed a significant intragroup increase following TAVR (Figure 4), whereas 8-isoprostane concentrations showed a significant intragroup increase following SAVR (Figure 5). FRAP was significantly higher in the TAVR group at the S2 interval, whilst 8-isoprostane concentrations were significantly higher in the SAVR group at the S3 interval. No statistically significant associations were observed between changes in erythrocytic glutathione status and circulating 8-isoprostane concentrations (data not shown).

The effect of red blood cell transfusion and procedural time variables following AVR was explored using a mixed model that included the treatment option and duration of the procedures adjusted by the STS score. Changes in ROS did not correlate with red blood cell transfusion and procedural time variables.

In-hospital and 3-month outcomes are summarized in Tables 5 and 6. There was no in-hospital death. Two patients (3.33%) developed ischemic stroke after the procedure, one in each group. VARC-2 defined major bleeding occurred in one patient undergoing TAVR and 2 patients undergoing SAVR. Patients undergoing SAVR received more transfusions, had longer ICU and overall hospital stay, and developed postprocedural atrial fibrillation more frequently than those treated with TAVR. The rate of myocardial infarction and acute renal failure was similar among the two groups. At 3-month follow-up, two patients had died in the TAVR group: one due to congestive heart failure and the second due to severe liver failure.

## 4. Discussion

The present study demonstrates that TAVR does not induce an acute oxidative stress response; this finding may be of particular significance for aged and frail patients at a high risk for periprocedural complications. Our data confirm and extend earlier findings using an electrochemical method [6] and are consistent with previous literature regarding the oxidative stress response in patients undergoing SAVR.

Oxidative stress was originally defined as an imbalance between ROS generation and antioxidant defense; over the years, the concept evolved further to reflect the complexity of the underlying regulatory processes and molecules involved and provide an explanation for the aberrations in redox regulation typically associated with pathophysiology [13, 14]. Under normal physiological conditions, ROS serve as integral components of cellular signaling pathways [14, 15]. A balanced redox state is established as a consequence of chemical and enzymatic interactions between the major ROS producing systems (NADPH oxidase, xanthine oxidase, nitric oxide synthase, myeloperoxidase, and lipoxygenases) and the major antioxidant systems (catalase, superoxide dismutase, glutathione peroxidase, and glutathione S transferases as well as *α*-tocopherol, ascorbic acid, reduced glutathione, and protein thiols) [14, 15]. Excess production or reduced degradation of ROS by the antioxidant defense systems imposes an oxidative burden upon the cellular environment leading to modification of various biomolecules and functional defects. In MRI, xanthine oxidase catalyzes the formation of uric acid with the coproduction of superoxide; superoxide release results in the recruitment and activation of neutrophils and their adherence to endothelial cells, which stimulates the formation of xanthine oxidase in the endothelium with further superoxide production [16]. Oxidation of DNA and proteins is accompanied by membrane damage initiated by lipid peroxidation, alterations in membrane permeability, modification of protein structures, and functional changes [17].

Patients with severe AS have been reported to have imbalances between endogenous oxidant and antioxidant characteristic of oxidative stress. This in turn has been linked to the pathogenesis of aortic valve degeneration [3, 18, 19]. One of the main advantages of TAVR is the relatively short ischemic time, which could mitigate MRI. We find that intracellular glutathione concentrations are significantly lower in patients undergoing TAVR, which may reflect a compromised capacity to cope with oxidative stress. Given that this observation remained significant after adjustment for the STS risk score, it would suggest that other confounders such as the patients' nutritional status and age may be important contributing factors.

We find an absence of an early oxidative stress response in patients undergoing TAVR as evidenced by changes in the concentration of 8-isoprostanes, a specific and reliable indicator of lipid oxidation. Unexpectedly, TAVR patients also presented with a significant and consistent increase in FRAP. This assay is independent of the availability of sulfhydryl groups, therefore complementary to the glutathione measurements in red blood cells, and often associated with low-molecular-weight antioxidants such as plasma uric acid. The increase in FRAP we observed may be related in part to the less invasive nature of TAVR, its lower ischemic time, and therefore a lesser MRI burden. Alternatively, the relatively rapid hemodynamic improvement following AVR may lead to enhanced shear stress-induced release of endothelial nitric oxide, which in addition to its role as an endogenous vasodilator has potent antioxidant properties [20, 21], possibly leading to an antioxidant-sparing effect. It may also indicate that the oxidative imbalance observed in patients with severe AS is amenable to correction by a minimally invasive and rapid restoration of normal physiology following relief of aortic obstruction.

Red blood cell storage has been linked to ROS generation and antioxidant consumption [22, 23]. It has been proposed that free radical-mediated damage may initiate and further exacerbate iron release during RBC storage further enhancing free radical-mediated cellular damage. In this study, we could not demonstrate any correlation between red blood cell transfusion and changes in oxidative stress biomarkers.

## 5. Study Limitations

Due to the nonrandomized nature of this observational study, the SAVR and TAVR groups were different with differing risk scores. Given the relatively small sample size, we did neither test for possible associations between any of the oxidative stress readouts with clinical outcomes nor perform any subgroup analyses. Rather, the aim in our study was to describe the temporal changes in ROS/oxidative stress in patients undergoing TAVR by using patients undergoing SAVR as a reference arm.

## 6. Conclusion

As compared to patients undergoing SAVR, patients undergoing TAVR did not show significant changes in biomarkers of oxidative stress despite having greater comorbidities and impaired baseline antioxidant defenses. TAVR was associated with an improvement in the antioxidant capacity of plasma. Larger studies would be required to determine if these potentially beneficial alterations are associated with clinical outcomes in patients undergoing TAVR.

## Figures and Tables

**Table 1:** Baseline clinical characteristics.

	TAVR (*n* = 38)	SAVR (*n* = 22)	*P* value
Age, years ± SD	79.3 ± 9.5	74.2 ± 4.1	0.02
Female, *n* (%)	25 (65.8)	9 (40.9)	0.06
Caucasian, *n* (%)	17 (44.7)	8 (36.4)	0.47
BMI, kg/m^2^ ± SD	27.6 ± 5.9	28.2 ± 3.8	0.67
Hypertension, *n* (%)	30 (78.9)	17 (77.3)	0.88
Type-2 diabetes, *n* (%)	15 (39.5)	3 (13.6)	0.03
Previous smoking, *n* (%)	17 (44.7)	3 (13.6)	0.01
Hypercholesterolemia, *n* (%)	16 (42.1)	8 (36.4)	0.66
Previous myocardial infarction, *n* (%)	11 (28.9)	0	0.01
Coronary artery disease	17 (44.7)	4 (9.1)	0.04
1-vessel CAD	4	4	
2-vessel CAD	7	0	
3-vessel CAD	6	0	
Previous PCI, *n* (%)	6 (15.8)	2 (9.1)	0.46
Previous CABG, *n* (%)	11 (28.9)	0	0.01
Previous CVA/TIA, *n* (%)	3 (7.9)	3 (13.6)	0.47
Peripheral vascular disease, *n* (%)	5 (13.2)	4 (18.2)	0.61
Chronic lung disease, *n* (%)	9 (23.7)	2 (9.1)	0.16
Chronic renal insufficiency	22 (57.9)	7 (31.8)	0.05
History of atrial fibrillation, *n* (%)	14 (36.8)	3 (13.6)	0.05
eGFR (mL/min)	59.8 ± 28.5	71.2 ± 019.2	0.10
Hemoglobin (g/dL)	12.0 ± 2.9	12.7 ± 1.6	0.26
STS risk score, ±SD	6.6 ± 4.8	3.2 ± 3.0	<0.01
Logistic Euroscore 2, ±SD	12.7 ± 8.8	5.1 ± 7.9	<0.01

BMI = body mass index; CAD = coronary artery disease; CABG = coronary artery bypass grafting; CVA = cerebrovascular accident; CHF = congestive heart failure; eGFR = estimated glomerular filtration rate; PCI = percutaneous coronary intervention; SD = standard deviation; STS = Society of Thoracic Surgeons; TAVR = transcatheter aortic valve replacement; TIA = transient ischemic attack.

**Table 2:** Baseline echocardiographic characteristics.

	TAVR (*n* = 38)	SAVR (*n* = 22)	*P* value
LV end-diastolic dimension, mm ± SD	48.3 ± 8.9	45.3 ± 5.8	0.25
LV end-systolic dimension, mm ± SD	33.5 ± 10.7	28.6 ± 7.9	0.14
LV ejection fraction, % ± SD	54.3 ± 18.0	61.2 ± 8.2	0.11
Aortic valve area, cm^2^ ± SD	0.6 ± 0.2	0.7 ± 0.3	0.30
Peak aortic velocity, m/s ± SD	4.4 ± 1.2	5.1 ± 1.1	0.16
Mean aortic valve gradient, mmHg ± SD	44.5 ± 15.9	51.8 ± 20	0.15
Pulmonary artery pressure, mmHg ± SD	46.6 ± 14.1	42.9 ± 17	0.55
Moderate to severe aortic regurgitation, *n* (%)	6 (15.8)	1 (4.5)	0.19
Moderate to severe mitral regurgitation, *n* (%)	8 (21.1)	2 (9.1)	0.23
Moderate to severe tricuspid regurgitation, *n* (%)	8 (21.1)	1 (4.5)	0.08

LV = left ventricle; SAVR = surgical aortic valve replacement; SD = standard deviation; TAVR = transcatheter aortic valve replacement.

**Table 3:** Procedural characteristics.

	TAVR (*n* = 38)	SAVR (*n* = 22)	*P* value
*Access site*			
Transfemoral, *n* (%)	28 (73.7)		
Transapical, *n* (%)	10 (26.3)		
Sternotomy, *n* (%)		100	
*Valve type*			
Sapien XT, *n* (%)	21 (79.4)		
Sapien 3, *n* (%)	11 (38.2)		
Evolut-R, *n* (%)	6 (47.1)		
Surgical bioprosthesis, *n* (%)		22 (100)	
Mean size, mm ± SD	25.4 ± 2.5	24.1 ± 2.2	0.07
Procedural time, min ± SD	99 ± 42	184 ± 43	<0.01
Fluoroscopy time, min ± SD	17.6 ± 7.9	—	
Cardiopulmonary bypass time, min ± SD	—	74.5 ± 31.6	
Aortic clamp time, min ± SD	—	61.0 ± 31.3	
General anaesthesia, *n* (%)	33 (86.8)	22 (100)	0.14
Conscious sedation, *n* (%)	5	0	

SAVR = surgical aortic valve replacement; SD = standard deviation; TAVR = transcatheter aortic valve replacement.

**Table 4:** Changes in oxidative stress-related biomarkers according to transcatheter or surgical aortic valve replacement.

Measured parameter mean ± SD	TAVR (*n* = 38)	SAVR (*n* = 22)	Adjusted *P* value
GSH S1	3.15 ± 0.79	3.57 ± 0.87	0.018
GSH S2	3.10 ± 0.90	2.87 ± 0.98	0.931
GSH S3 (nmol/mg protein)	3.23 ± 0.74	2.85 ± 0.77	0.498
Slope for trend	0.022	-0.330	
*P* value	0.698	<0.001	
GSSG S1	0.27 ± 0.18	0.29 ± 0.11	0.942
GSSG S2	0.30 ± 0.32	0.39 ± 0.32	0.650
GSSG S3 (nmol/mg protein)	0.26 ± 0.15	0.30 ± 0.13	0.475
Slope for trend	-0.005	0.003	
*P* value	0.842	0.804	
GSH/GSSG S1	16.5 ± 9.1	13.9 ± 5.1	0.568
GSH/GSSG S2	15.4 ± 9.3	10.9 ± 7.7	0.380
GSH/GSSG S3	20.5 ± 27.4	11.8 ± 6.5	0.237
Slope for trend	1.860	-0.880	
*P* value	0.319	0.199	
FRAP S1	339.1 ± 90.9	329.8 ± 74.1	0.410
FRAP S2	359.6 ± 94.3	314.8 ± 68.6	0.017
FRAP S3 (*μ*mol/L)	357.0 ± 119.8	327.1 ± 113.5	0.212
Slope for trend	12.6	-0.22	
*P* value	0.027	0.983	
8-Isop S1	40.6 ± 18.3	41.0 ± 16.1	0.553
8-Isop S2	41.0 ± 22.1	41.8 ± 22.3	0.635
8-Isop S3 (pg/mL)	40.1 ± 21.8	52.0 ± 26.1	0.046
Slope for trend	-2.56	46.5	
*P* value	0.86	0.028	

GSH = reduced glutathione; GSSG = oxidized glutathione; FRAP = ferric-reducing ability of plasma; S = sample; SAVR = surgical aortic valve replacement; SD = standard deviation; TAVR = transcatheter aortic valve replacement; 8-Isop = 8-isoprostane.

**Table 5:** In-hospital clinical outcomes.

	TAVR (*n* = 38)	SAVR (*n* = 22)	*P* value
Death, *n* (%)	0	0	0.99
Cerebrovascular accident, *n* (%)	1 (2.6)	1 (4.5)	0.69
Myocardial infarction, *n* (%)	0	0	0.99
Major bleeding, *n* (%)	1 (2.6)	2 (9.1)	0.27
Periprocedural transfusions, *n* (%)	1 (2.6)	8 (36.4)	<0.01
Red blood cell transfusion, units ± SD	0.1 ± 0.3	0.6 ± 1.7	0.06
New atrial fibrillation, *n* (%)	3 (7.9)	6 (27.3)	0.04
Permanent pacemaker, *n* (%)	2 (5.3)	2 (9.1)	0.57
Acute renal failure, *n* (%)	3 (7.9)	3 (9.1)	0.87
Stay at intensive care unit, hours ± SD	64 ± 65	128 ± 130	0.01
Length of admission, days ± SD	4.9 ± 2.6	8.4 ± 4.6	<0.01

SAVR = surgical aortic valve replacement; SD = standard deviation; TAVR = transcatheter aortic valve replacement.

**Table 6:** Clinical outcomes at 90-day follow-up.

	TAVR (*n* = 38)	SAVR (*n* = 22)	*P* value
Death, *n* (%)	2 (5.3)	0	0.27
Readmission, *n* (%)	3 (7.9)	1 (4.5)	0.62
Cerebrovascular accident, *n* (%)	1 (2.6)	1 (4.5)	0.68
Myocardial infarction, *n* (%)	0	0	0.99

SAVR = surgical aortic valve replacement; TAVR = transcatheter aortic valve replacement.

## Data Availability

The data used to support the findings of this study are available from the corresponding author upon request.

## Abbreviations

AS: Aortic stenosis
FRAP: Ferric-reducing ability of plasma
GSH: Reduced glutathione
GSSH: Oxidized glutathione
MRI: Myocardial ischemia and reperfusion injury
ROS: Reactive oxygen species
SAVR: Surgical aortic valve replacement
STS: Society of Thoracic Surgeons
TAVR: Transcatheter aortic valve replacement.
